# Supplementary material for: Chloroplast–mitochondria synergy modulates responses to iron limitation in two Thalassiosira diatom species
Source: ISME Commun. 2026 Aug 19;6(1):ycag236. doi: 10.1093/ismeco/ycag236 (PMC13588709; doi:10.1093/ismeco/ycag236)
Supplement: Supplementary file 1 [file web_material_ycag236.zip › fig_S1_260811_ycag236.pdf]

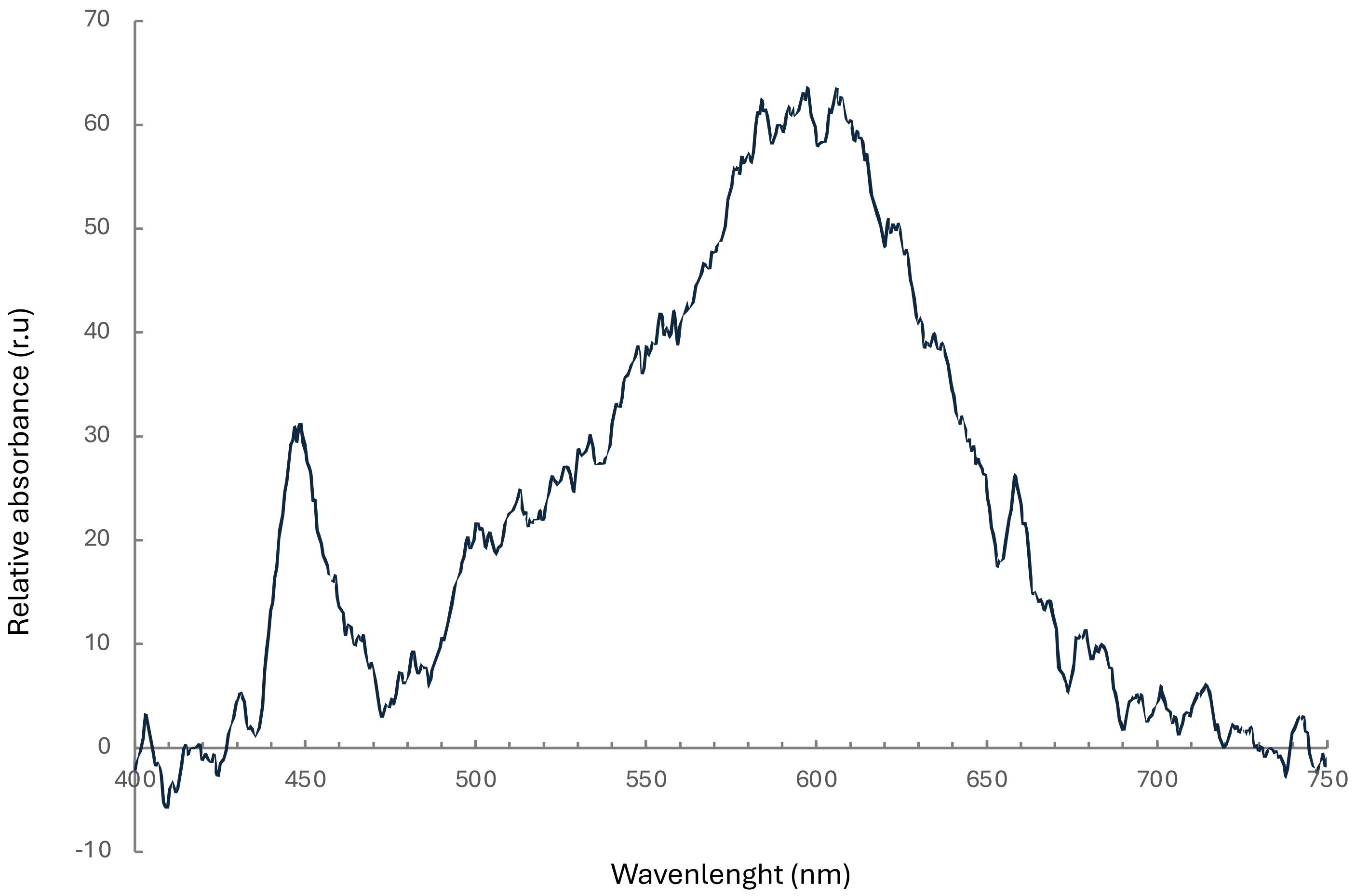

Figure S1. Spectrum of the incident light used in the growth incubator. Measured with an AvaSpec spectrophotometer UV-Vis-NIR (Avantes)against dark.
