# Supplementary material for: Chloroplast–mitochondria synergy modulates responses to iron limitation in two Thalassiosira diatom species
Source: ISME Commun. 2026 Aug 19;6(1):ycag236. doi: 10.1093/ismeco/ycag236 (PMC13588709; doi:10.1093/ismeco/ycag236)
Supplement: Supplementary file 1 [file web_material_ycag236.zip › fig_S2_revised_ycag236.pdf]

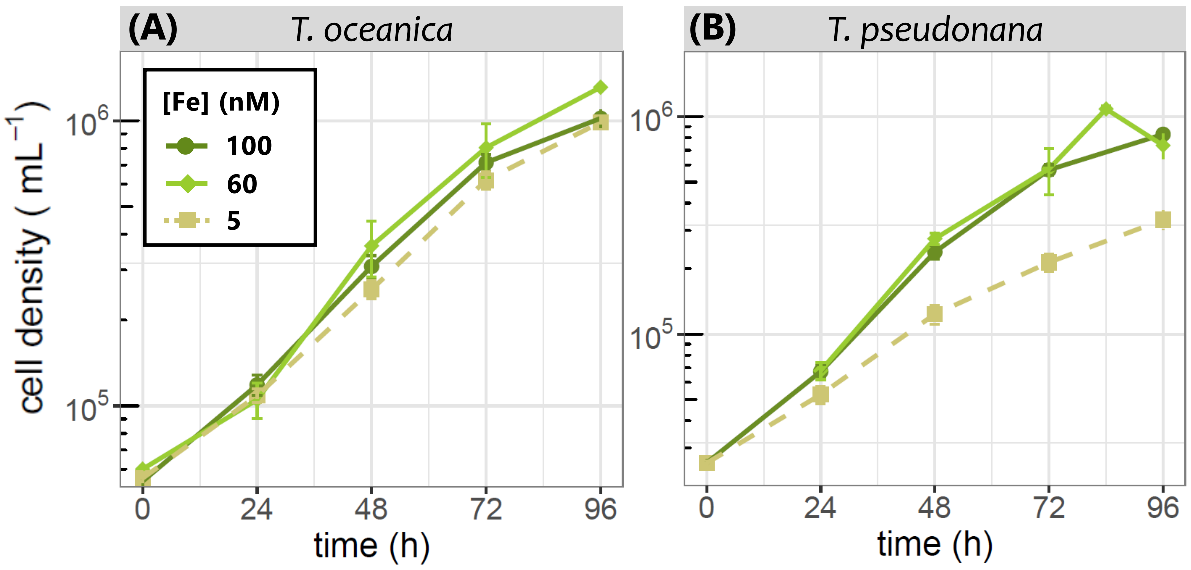

Figure S2. Effect of Fe concentration on *Thalassiosira sp*. growth in ASW-based media*:* Cultures of *T. oceanica* **(A)** and *T. pseudonana* **(B)** were inoculated at an initial cell density of $6{\times}10^4$ and $3{\times}10^4$ cells/mL respectively, from refreshed and acclimated cultures, into L1 ASW-based medium supplemented with different total Fe concentrations. The curves are representative of at least 2 replicates. Error bars indicate standard deviation when applicable.
