# Supplementary material for: Chloroplast–mitochondria synergy modulates responses to iron limitation in two Thalassiosira diatom species
Source: ISME Commun. 2026 Aug 19;6(1):ycag236. doi: 10.1093/ismeco/ycag236 (PMC13588709; doi:10.1093/ismeco/ycag236)
Supplement: Supplementary file 1 [file web_material_ycag236.zip › Table_S1_ycag236.docx]

| **Species** | **Iron conditions** | **Replicate ID** | **Mg** | **P** | **K** | **Mn** | **Fe** | **Cu** | **Zn** |
| --- | --- | --- | --- | --- | --- | --- | --- | --- | --- |
| *T. oceanica* | **+Fe** | **#1** | 3.92E-05 | 9.84E-05 | 2.26E-04 | 1.18E-07 | 1.28E-06 | 4.92E-08 | 7.07E-07 |
| *T. oceanica* | **+Fe** | **#2** | 7.42E-05 | 1.79E-04 | 4.58E-04 | 3.50E-07 | 2.89E-06 | 4.91E-08 | 7.42E-07 |
| *T. oceanica* | **+Fe** | **#3** | 8.20E-05 | 1.81E-04 | 4.26E-04 | 5.07E-07 | 3.46E-06 | 6.25E-08 | 1.02E-06 |
| *T. oceanica* | **-Fe** | **#1** | 5.31E-05 | 1.14E-04 | 2.42E-04 | 2.44E-07 | 2.18E-06 | 4.17E-08 | 7.64E-07 |
| *T. oceanica* | **-Fe** | **#2** | 6.01E-05 | 1.51E-04 | 2.95E-04 | 2.69E-07 | 2.10E-06 | 4.51E-08 | 7.72E-07 |
| *T. oceanica* | **-Fe** | **#3** | 5.56E-05 | 1.49E-04 | 2.95E-04 | 2.71E-07 | 2.34E-06 | 5.06E-08 | 8.51E-07 |
| *T. pseudonana* | **+Fe** | **#1** | 2.82E-04 | 2.10E-04 | 2.18E-04 | 1.03E-06 | 1.52E-05 | 1.39E-07 | 5.42E-06 |
| *T. pseudonana* | **+Fe** | **#2** | 3.36E-04 | 1.66E-04 | 2.17E-04 | 1.24E-06 | 2.06E-05 | 1.22E-07 | 2.06E-06 |
| *T. pseudonana* | **+Fe** | **#3** | 5.12E-05 | 1.21E-04 | 1.97E-04 | 5.12E-07 | 3.62E-06 | 6.07E-08 | 7.92E-07 |
| *T. pseudonana* | **-Fe** | **#1** | 1.17E-04 | 4.65E-04 | 6.13E-04 | 1.62E-06 | 3.60E-06 | 2.37E-07 | 1.10E-06 |
| *T. pseudonana* | **-Fe** | **#2** | 4.07E-04 | 2.10E-04 | 3.08E-04 | 1.67E-06 | 1.67E-05 | 5.11E-07 | 3.35E-06 |
| *T. pseudonana* | **-Fe** | **#3** | 2.72E-04 | 1.27E-04 | 2.44E-04 | 9.09E-07 | 1.29E-05 | 1.33E-07 | 1.72E-06 |

**Table S1: Elemental analysis of diatoms grown under iron-replete or iron-limited conditions.** *T. oceanica* and *T. pseudonana* cells were grown under iron-rich (+Fe) and iron-poor (-Fe) conditions (3 independent biological replicates). Cells were washed three times in 0.3 M NaCl, 10 mM KCl, 20 mM HEPES pH 7.0, then dried and digested in ultrapure HNO₃. The concentrations of Mg, P, K, Mn, Fe, Cu and Zn in the mineralizates (mol·L⁻¹) were determined by ICP-MS.
